# Utility of pharmacogenetic testing to optimise antidepressant pharmacotherapy in youth: a narrative literature review

**DOI:** 10.3389/fphar.2023.1267294

**Published:** 2023-09-19

**Authors:** Bradley Roberts, Zahra Cooper, Stephanie Lu, Susanne Stanley, Bernadette T. Majda, Khan R. L. Collins, Lucy Gilkes, Jennifer Rodger, P. Anthony Akkari, Sean D. Hood

**Affiliations:** ^1^ The Perron Institute for Neurological and Translational Science, Nedlands, WA, Australia; ^2^ School of Biological Sciences, University of Western Australia, Crawley, WA, Australia; ^3^ School of Psychological Science, University of Western Australia, Crawley, WA, Australia; ^4^ Division of Psychiatry, School of Medicine, University of Western Australia, Crawley, WA, Australia; ^5^ School of Medicine, University of Notre Dame, Fremantle, WA, Australia; ^6^ Western Australian Department of Health, North Metropolitan Health Service, Perth, WA, Australia; ^7^ Divison of General Practice, School of Medicine, University of Western Australia, Crawley, WA, Australia; ^8^ School of Human Sciences, University of Western Australia, Crawley, WA, Australia; ^9^ Centre for Molecular Medicine and Innovative Therapeutics, Murdoch University, Murdoch, WA, Australia; ^10^ Division of Neurology, Duke University Medical Centre, Duke University, Durham, United States

**Keywords:** pharmacogenetics, pharmacogenomics, personalised medicine, cytochrome P450, drug metabolism, youth mental health, depression, anxiety

## Abstract

Pharmacogenetics (PGx) is the study and application of how interindividual differences in our genomes can influence drug responses. By evaluating individuals’ genetic variability in genes related to drug metabolism, PGx testing has the capabilities to individualise primary care and build a safer drug prescription model than the current “one-size-fits-all” approach. In particular, the use of PGx testing in psychiatry has shown promising evidence in improving drug efficacy as well as reducing toxicity and adverse drug reactions. Despite randomised controlled trials demonstrating an evidence base for its use, there are still numerous barriers impeding its implementation. This review paper will discuss the management of mental health conditions with PGx-guided treatment with a strong focus on youth mental illness. PGx testing in clinical practice, the concerns for its implementation in youth psychiatry, and some of the barriers inhibiting its integration in clinical healthcare will also be discussed. Overall, this paper provides a comprehensive review of the current state of knowledge and application for PGx in psychiatry and summarises the capabilities of genetic information to personalising medicine for the treatment of mental ill-health in youth.

## 1 Introduction

Interindividual differences in our genomes occur within and between demographic populations, leading to genomic variation and individual phenotype presentation (Vallejos-Vidal et al., 2019). Variations in genes associated with drug absorption and metabolism may have significant effects on a person’s response to medication. Understanding these genetic variations has the potential to improve drug selection and dosage in clinical practice and may ultimately better the quality of medical care (Theoktisto et al., 2009; Wall et al., 2010). The study of variations within those genes involved in drug metabolism and how they influence an individual’s biological response to medication is known as “pharmacogenetics” (PGx) (Pirmohamed, 2001); a field of research showing promise as an interventional application for personalised and precision medicine (Roden and Tyndale, 2013; Zgheib and Patrinos, 2020).

The factors affecting drug efficacy and toxicity can have significant consequences for patient health outcomes and contribute to personal and national economic loss associated with avoidable hospital admission. In regard to the United States Food and Drug Administration’s (FDA) top ten highest-grossing drugs, for every person experiencing a therapeutic benefit, it is estimated that between three and 24 individuals fail to show any response (Schork, 2015). A recent review on PGx perspectives quoted Allen Roses, MD, stating: “The vast majority of drugs—more than 90%—only work in 30% or 50% of people” (Pirmohamed, 2023). Side effects and adverse events to medication are believed to account for ∼6.5% of hospital admissions (Pirmohamed et al., 2004; Soiza, 2020), with Australia alone reporting that ∼7.2% of all medical admissions are medication related (Roughead et al., 2016). Though not the only contributing factor, four out of five people are believed to carry genetic variations that may alter drug efficacy and safety (Schärfe et al., 2017), with some experts estimating genetic factors to account for up to 95% of treatment response (Oates and Lopez, 2018). Furthermore, patient risk of adverse events and serious side effects from many drugs varies with respect to geographical ancestry (Shah and Gaedigk, 2018). The application of PGx aims to move drug prescription away from the current “one-size-fits-all” model to a more personalised and precise manner of medication choice tailored to each individual (Pirmohamed, 2023).

Mental health disorders represent a significant proportion of the burden of disease in youth worldwide (Malhi and Mann, 2018). People under the age of 24 years are particularly vulnerable to mental illness, with 30%–50% of this age group suffering from depressive and anxiety disorders non-responsive to cognitive behavioural therapy and primary medication-based treatment (Kessler et al., 2005; Lefaucheur et al., 2020). Research shows that an untreated mental health condition in younger years can have chronic, long-lasting effects, shaping the lives of young people into adulthood (Goodsell et al., 2017). Furthermore, a rise in mental health prevalence in recent years has caused a dramatic increase in the use and prescription rates of antidepressants (de Oliveira Costa et al., 2023). Between 2015 and 2019, de Olivera Costa et al. reported over 50% of young Australian males and females aged between 10 and 17 years received a new antidepressant prescription. Given the rising incidence and the stark increase in psychiatric medication use, there is an urgent need to ensure young people with mental health issues receive effective care. Guiding antidepressant selection and dosage with PGx testing has been suggested to improve mental health outcomes and limit adverse medication-related events (Bousman et al., 2019).

This review comprises three sections. The first section presents an overview of PGx and its role in clinical practice, with a particular focus on the role of cytochrome P450 (CYP) drug-metabolizing enzymes. The second section investigates the involvement of CYP enzymes in the metabolism of antidepressant medication and the use of PGx in improving mental health outcomes in randomized clinical trials. The third section addresses the application of PGx testing in youth mental health, evaluating the existing barriers hindering the implementation of PGx in primary healthcare. It explores the gaps in the literature pertaining to PGx in psychiatric care and provides recommendations for future research required to progress PGx into routine clinical practice.

## 2 Pharmacogenetics

### 2.1 What is Pharmacogenetics and Pharmacogenomics?

PGx comes under the umbrella term “pharmacogenomics”, encompassing the understanding of how an individual’s genetic makeup (genotype) may influence their metabolic status (phenotype) and overall response to a drug, in the context of both efficacy and toxicity (Pirmohamed, 2023). Pharmacogenomics includes two main determinants of drug response, pharmacodynamics and pharmacokinetics, with pharmacodynamics studying the drug’s effect on the body and pharmacokinetics studying the body’s effects on the drug (Adams, 2008). More specifically, pharmacodynamics is the study of a drug’s effects both biochemically and physiologically on its molecular target, observing the downstream effects that are elicited by the drug-target interaction (Marino et al., 2023). In contrast, pharmacokinetics is the study of how the body interacts with and affects the elicited outcome of an administered substance such as a pharmaceutical drug (Grogan and Preuss, 2023). The four main components of pharmacokinetics are the processes of absorption, distribution, metabolism, and excretion, which are all affected by an individual’s genetics. Analysing the variations present in one’s genes affecting these processes (PGx testing), allows us to better understand how their body may respond to different medication at varying doses. Thus, PGx testing has become a way to adapt and optimise drug prescription and dosing in a clinical environment, by predicting the interactions between pharmacokinetic parameters and genetic variability.

### 2.2 The role of cytochrome P450 enzymes in pharmacogenetic variation

**FIGURE 1:**
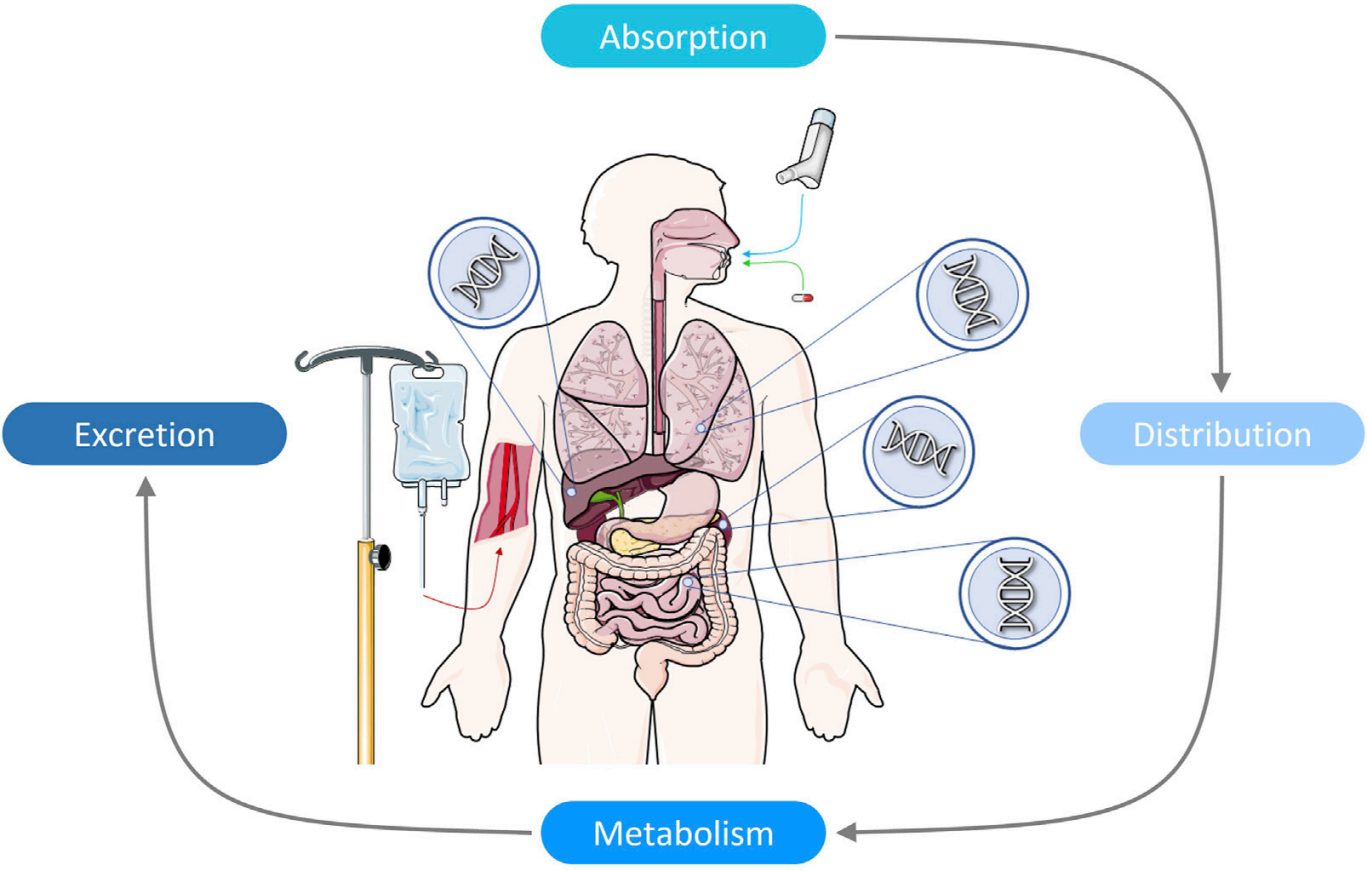
Pharmacogenetics is the study of how our individual genetic makeup can affect the properties of pharmacokinetics and pharmacodynamics. More specifically, how our genetics can influence our body’s absorption, distribution, metabolism, and excretion of medication for all health conditions. Variation in genes encoding drug metabolizing enzymes, transporters, and/or pharmacological receptors found predominantly in the liver, but also found in the lungs, kidneys, and small intestine, can provide clinical information on a person’s drug-metabolic status, and may predict how they will respond to pharmacotherapy.

Genetics influences pharmacodynamic and pharmacokinetic properties. In particular, affecting how a drug is absorbed, distributed, metabolised, and excreted in an individual, all factors contributing to drug efficacy and tolerability (Figure 1) (Kalow, 1990; Weinshilboum, 2003; Horstmann and Binder, 2009). Variation in genes involved in drug response (PGx genes) is common within the human population, with a recent review providing evidence that 97.8% of people worldwide are likely to carry an actionable genetic variant in a PGx gene (Pirmohamed, 2023). Furthermore, over half the drugs currently prescribed in clinical practice are metabolised by PGx genes and are affected by one or more PGx variants (Dunnenberger et al., 2015; Kimpton et al., 2019).

Variations in genes encoding drug metabolizing enzymes, transporters, and/or pharmacological receptors have been shown to have a profound impact on an individual’s drug response (Shah, 2005). Involved in more than 90% of all enzymatic reactions, CYP enzymes catalyse iron-induced oxidation reactions to convert lipid-soluble compounds to more water-soluble compounds for excretion (Guengerich, 2018). CYPs are a family of genes that encode enzymes responsible for key roles in the metabolism of drugs and other foreign compounds (i.e., xenobiotics). CYPs are some of the most diverse catalysts in biochemistry and contribute to large intraindividual variability in the safety, efficacy, and tolerability of drug response (Zhao et al., 2021).

CYP gene sequences are classified by a family number, subfamily letter, and then at the protein level further differentiated by isoform number (e.g., *CYP2C9*, *CYP1A2*, *etc.*) (McDonnell and Dang, 2013). Currently there are 57 known CYP genes with just six CYP genes encoding enzymes that metabolise ∼90% of all drugs (Lynch et al., 2007). A number of CYP genes have functional polymorphisms, yielding enzyme isoforms that vary in their ability to metabolise drugs, therefore influencing the biologically available concentration of the active drug compound (Turner, 2013). Recognizing how these polymorphisms affect drug tolerability and which individuals carry these mutations is important when trying to understand and predict how individual alleles may influence drug-drug interactions, the likelihood of adverse events, and drug therapeutic outcomes. For example, a recent study by Koopmans et al. (2021) suggests that ∼36% of people worldwide are likely to at least one actionable variant in the *CYP2D6* enzyme-encoding gene, and ∼62% are likely to one in the *CYP2C19* encoding gene, altering their metabolic efficiency of drugs digested by these enzymes. Through PGx testing, an individual’s drug metabolic phenotype can be classified into four main subtypes: poor metabolisers, intermediate metabolisers, extensive metabolisers, or ultrarapid metabolisers (Bousman et al., 2019). This information can help clinicians make informed decisions on drug prescription and dosing for each patient based on known personalised genetic data, rather than average population statistics. As these variations are encoded in the human genome, PGx testing is suggested as an efficient and effective tool to tailor prescription medication to patients.

Considerable CYP allelic carriage differences are not only seen between individuals but have previously been noted between people of varying ethnic backgrounds (McGraw and Waller, 2012; Shah and Gaedigk, 2018). For example, a higher proportion of poor metabolisers of *CYP2C19* has been reported in Asian populations (∼15–30%) compared to populations of European, African, and Arab decent (∼3–6%) (Poolsup et al., 2000). Similarly, the proportion of poor metabolisers of the *CYP2D6* gene appears to be higher in Europeans and Caucasian Americans (∼7%) than in Asian populations (∼1%). Differences in CYP allele carriage such as these may possibly lead to large variances in clinical drug responses between ethnically diverse groups, disadvantaging minority backgrounds (Poolsup et al., 2000).

Allelic variation in CYPs is not likely due to *de novo* mutations, but more likely to provide advantageous evolutionary traits (van der Weide and Hinrichs, 2006). Although genetic influence and inherited attributes are considered key contributors to drug response (Xie et al., 2001; Murphy et al., 2013; Shah, 2015), research suggests that drug-metabolising enzymes have evolved due to our interaction with our environment. Diet then, is likely as important as genetic ancestry when understanding a person’s response to medication (Wilson et al., 2001). For instance, foods, including fruits, alcohol, teas, and herbs are commonly known to induce or inhibit the activity of drug metabolising enzymes, such as CYPs (Fujita, 2004). For example, St John’s wort (*Hypericum perforatum*) extract has long been associated with relieving the symptoms of mild depression, demonstrating a high safety and efficacy profile (Canenguez Benitez et al., 2022). However, research has shown that St John’s wort is a potent inducer of CYP enzyme CYP3A4, resulting in the enhanced metabolism and decreased blood concentration of drugs metabolised by this enzyme, including common antidepressants citalopram, fluoxetine, and sertraline (Zhou et al., 2004). Because of this interaction, St John’s wort is a major safety concern when consumed in combination with antidepressants.

These considerations show that whilst PGx testing to guide treatment is important for the integration of precision medicine into primary care, the discussion of the results of genetic testing should not disregard a patient’s ethnicity and cultural background when used to prescribe medication (Shah and Gaedigk, 2018).

### 2.3 Pharmacogenetic testing in clinical practice

PGx testing has been commercially available for nearly two decades, yet its implementation into clinical practice has been slow. Currently, PGx testing has been restricted to a few specialist clinics at the forefront of the field, predominantly in North America and Europe^1^ (Volpi et al., 2018; Haidar et al., 2019; Maruf et al., 2020; Smith et al., 2020). The lack of adoption is surprising as PGx testing has been demonstrated in clinical trials to significantly increase the efficacy and tolerability of prescribed drugs (Pirmohamed et al., 2013). The most commonly prescribed drugs with FDA-approved PGx recommendations are anti-inflammatory medication, anti-blood clotting medication, and pain relief medication (Smith et al., 2020).

In addition to improving safety and efficacy, the use of PGx in clinical practice has the potential to reduce costs to both the individual and the economy. In Australia, it is estimated that ∼400,000 patients are admitted to hospital emergency departments with adverse drug reactions (ADRs) related to their prescription medication (Phillips et al., 2014). By current estimates, Australian healthcare expenditure for ADRs is estimated at AUD ∼$1.4 billion per annum^2^. However, only 6% of all ADRs that occur are reported, suggesting that the real cost to the Australian economy is substantially higher (Bailey et al., 2016). Incorporating routine PGx testing into clinical use would likely reduce the frequency and severity of ADRs, minimise the prescription of ineffective medications and streamline treatment (Verbelen et al., 2017; Swen et al., 2023). Furthermore, with the availability of simple buccal swab genetic testing, PGx-guided treatment has become an accessible form of personalised medicine at relatively low cost to patients (Blumberger et al., 2018). The incorporation of PGx testing into clinical practice is integral for the promotion of patient-centered care and addressing populace health disparities by ensuring that all patients receive the best possible care, regardless of their genetic background (Hicks et al., 2019).

## 3 Pharmacogenetics in the treatment for mental health

Mental health disorders are among the most prevalent health conditions in Western society (De Vaus et al., 2018). The World Health Organisation estimated 1 in 8 people were suffering from a diagnosable mental illness prior to the COVID-19 pandemic, with numbers rising by ∼27% in following years^3^. By 2030, mental health disorders are projected to be the first ranked cause of disease burden worldwide (Friedrich, 2017), with ∼1 in 5 people expected to experience an episode of mental ill-health at some stage in their lifetime (Malhi and Mann, 2018). Depression and anxiety are amongst the most common mental health disorders observed in general practice, often seen as comorbid conditions that are treated similarly by primary healthcare practitioners (Tiller, 2013). Psychological therapy is frequently used as a first port of call for treating patients with depression and anxiety at a mild to moderate severity, however, patients with a more severe condition often undergo psychological treatment in conjunction with pharmacotherapy. As most second-generation antidepressant medications also show promising results in reducing levels of anxiety (Cassano et al., 2002), both conditions can typically be treated through the prescription of one or more antidepressants (Ballenger, 2000).

### 3.1 Understanding the role of cytochrome P450s in psychiatry

Although antidepressants are the most frequently prescribed medication for mental health disorders (Lunghi et al., 2022), their efficacy in relieving symptoms of depression and anxiety are variable (Maslej et al., 2021). Whilst a considerable number of patients will undergo remission within 2 months of treatment, over half experience minimal improvement, with some patient’s depressive symptoms worsening (Thomas et al., 2013). It is believed that between ∼42–50% of this variability in antidepressant drug response is accounted for by genetic variation in PGx genes (Crisafulli et al., 2011; Tansey et al., 2013). Although there are over 50 CYP genes involved in drug metabolism (Ingelman-Sundberg et al., 2007; Lynch et al., 2007; Zhao et al., 2021), only a small number have major influences on the metabolism of antidepressants (Table 1) (Zemanova et al., 2022). Approximately 24% of antidepressants are metabolised by *CYP1A2*-encoded enzymes, 5% by *CYP2B6*, 38% by *CYP2C19*, 85% by *CYP2D6*, and 38% by *CYP3A4*, however, this may vary subject to age, sex, and ethnicity related differences (Cacabelos and Torrelas, 2015). Most antidepressants are metabolised by numerous CYPs, whilst it is possible for a single CYP to be involved in the metabolism of multiple different antidepressants (Radosavljevic et al., 2023). Variations in allelic carriage of these CYP enzyme-encoding genes is known to alter enzymatic activity and affect drug efficacy, therapeutic outcomes, and possible side effect presentation (van Westrhenen et al., 2021).

**TABLE 1:** Royal Australian and New Zealand College of Psychiatrists approved and recommended antidepressants, their primary mechanism of action and cytochrome P450 metabolic pathways. Minor metabolic pathways in parentheses (Cacabelos, 2012; Samer et al., 2013; Malhi et al., 2015; Malhi et al., 2021; Zemanova et al., 2022).

Drug class	Generic drug name	Cytochrome P450 metabolism	Primary mechanism of action	References
Monoamine Oxidase Inhibitors (MAOIs)	Moclobemide^a^	CYP2C19 (CYP1A2, CYP2D6)	Inhibits the oxidative deamination of monoamines A and B to increase concentrations of 5-HT, NA, and DA within the presynaptic neuron	Bonnet (2003); Bunney and Davis (1965); Gillman, (2011); Schildkraut, (1965); Sub Laban and Saadabadi (2022)
	Phenelzine	(CYP3A4, CYP2C19)		
	Tranylcypromine	CYP2A6	Last-line antidepressants due to irreversible nature of inhibition	
Tricyclic Antidepressants (TCAs)	Amitriptyline	CYP2C19, CYP3A4 (CYP1A2)	Diverse mechanisms of action though most inhibit the reuptake of NA and 5-HT to increase neurotransmitter concentrations in the synaptic cleft for postsynaptic uptake. High affinity for NA reuptake transporters over 5-HT. Some TCAs also act as DA receptor antagonists	Gillman (2007); Hillhouse and Porter (2015); Sheffler et al. (2022) ^c^
	Clomipramine	CYP2C19 (CYP1A2, CYP3A4)	Secondary antidepressants due to off-target effects caused by antagonism for postsynaptic histamine, muscarinic, and adrenergic receptors	
	Dosulepin	CYP2C19, CYP3A4, CYP1A2 (CYP2D6)		
	Doxepin	CYP2C19, CYP3A4, CYP1A2, CYP2C9 (CYP2D6)		
	Imipramine	CYP2C19 (CYP1A2)		
	Nortriptyline	CYP2D6 (CYP1A2, CYP2C19, CYP3A4)		
	Amoxapine^b^	CYP2D6, CYP1A2, CYP2C9, CYP2C19, CYP3A4		
Selective Serotonin Reuptake Inhibitors (SSRIs)	Citalopram	CYP2C19, CYP3A4 (CYP2D6)	Selectively inhibits 5-HT reuptake transporters, increasing 5-HT concentration in the synaptic cleft. Weak affinity for NA reuptake transporters	Castrén and Rantamäki (2010); Hillhouse and Porter (2015); Sangkuhl et al. (2009); Santarelli et al. (2003); Shaw et al. (1967); Wong et al. (1975); Wong et al. (1974); Castrén and Rantamäki (2010)
	Escitalopram	CYP2C19, CYP3A4, CYP2D6	Increases the expression of neurotrophic factors such as brain-derived neurotrophic factor (BDNF), enhancing hippocampal neurogenesis and neuroplasticity	
	Fluoxetine	CYP2D6, CYP2C9, CYP3A4 (CYP2C19)	Primary first-line treatment option due to their strong tolerability and safety profile	
	Fluvoxamine	CYP2D6 (CYP1A2)		
	Paroxetine	CYP2D6, CYP2B6		
	Sertraline	CYP2B6 (CYP2C19, CYP2C9, CYP3A4, CYP2D6)		
Serotonin-Noradrenaline Reuptake Inhibitors (SNRIs)	Desvenlafaxine	CYP3A4	Inhibits the reuptake of both NA and 5-HT with little to no off-target effects on histamine, muscarinic, and adrenergic receptors. Reuptake inhibition leads to an increase in prefrontal DA concentration	Bymaster et al. (2001); Liebowitz and Tourian (2010); Millan et al. (2001); Papakostas et al. (2007); Paris et al. (2009); Vaishnavi et al. (2004)
	Duloxetine	CYP2D6, CYP1A2		
	Milnacipran	CYP2B6, CYP3A4		
	Levomilnacipran	CYP3A4 (CYP2C8, CYP2C19, CYP2D6, CYP2J2)	Primary first-line treatment option	
	Venlafaxine	CYP2D6 (CYP2C19, CYP3A4)		
Selective Noradrenaline Reuptake Inhibitors (NRIs)	Reboxetine	CYP3A4	Selectively inhibits the reuptake of NA by the presynaptic membrane to increase prefrontal DA and NA levels without significantly affecting subcortical DA concentrations	Brunello et al. (2002); Bymaster et al. (2002); Dillon and Pizzagalli (2018); Nestler et al. (2002); Steiger and Pawlowski (2019)
	Atomoxetine	CYP2D6	Well tolerated first-line antidepressants used primarily for their stimulating effects such as cortical arousal and increased energy levels	
	Teniloxazine	?		
Serotonin Modulators	Trazodone	CYP3A4	High doses: inhibits 5-HT reuptake via 5-HT 2A/2C	Ellingrod and Perry (1995); Malhi et al. (2015); Rotzinger and Baker (2002); Wang et al. (2013)
			Low doses: antagonises 5-HT 2A, adrenergic, and histamine	
	Vortioxetine	CYP2D6, (CYP3A4, CYP2A4, CYP2C19, CYP2C9)	Inhibits 5-HT transporters, agonists for 5-HT1a and 1b, and antagonists for 5-HT3a and 7	
	Nefazodone	CYP3A4	Inhibits 5-HT transporters to increase cortical neurotransmitters (NA, DA, and BDNF)	
	Vilazodone	CYP3A4 (CYP2C19, CYP2D6)	Selective inhibitor for 5-HT reuptake and partial agonist on 5-HT1a receptors	
Atypical Antidepressants	Agomelatine	CYP1A2, (CYP2C9, CYP2C19)	First antidepressant to directly increase melatonin levels, acting as a melatonin agonist and 5-HT antagonist to further promote the increased release of both DA and NA.	Bymaster et al. (2002)
	Mirtazapine^b^	CYP2D6, CYP3A4, (CYP1A2)	Inhibits 5-HT reuptake transporters, antagonises adrenergic receptors to increase NA, and antagonises 5-HT to increase cortical NA and DA. Mirtazapine is commonly used as first-line treatment	Harmer et al. (2017); Hickie and Rogers (2011)
	Mianserin^b^	CYP2D6, CYP1A2 (CYP3A4)		
	Bupropion^b^	CYP2B6	Inhibits the reuptake of both NA and DA, prolonging their duration of action within the synaptic cleft and promoting downstream effects	Horst and Preskorn (1998)
	Maprotiline^b^	CYP2D6	Predominantly a NA and DA reuptake inhibitor but also antagonises histamine and adrenergic receptors. Maprotiline also acts as an inhibitor to amine transporters to delay NA reuptake	Llorca (2010); Montejo et al. (2010)
NMDA-Glutamatergic Receptor Antagonists	Esketamine	CYP2B6, CYP3A4 (CYP2C9, CYP2C19)	Selective antagonists of glutamate NMDA receptors that bind to the glutamate site of the receptor, thereby inhibiting the release of calcium into the neuron and increasing prefrontal and hippocampal glutamate concentrations	Edinoff et al. (2021)
	Ketamine	CYP2C9, CYP2B6, CYP3A4	Esketamine: non-competitive; ketamine: competitive	Lener et al. (2017); Lin et al. (2015); Singh et al. (2016) ^d^
	Brexanolone	Non-CYP based pathways	Enhances the inhibitory effects of GABA by antagonising GABA_A_ receptors to promote stimulation of glutamate production	
Atypical Antipsychotics	Aripiprazole	CYP2D6, CYP3A4	Acts as a partial agonist at D2 receptors acting on both postsynaptic DA2 receptors and presynaptic autoreceptors to enhance DA activity in the mesocortical pathway. Also modulates 5-HT2A receptors	Bauman et al. (2008); DeLeon et al. (2004); McNeil et al. (2022); Stahl (2016); Thomas and Saadabadi (2022); Zubiaur et al. (2021) ^e^
	Brexpiprazole	CYP2D6, CYP3A4		
	Lurasidone	CYP3A4	Antagonist for DA2 and 5-HT2A receptors to increase neurotransmitter concentrations and normalise brain activity	
	Quetiapine	CYP3A4 (CYP2D6)	Antagonists for 5-HT2A and D2 receptors, increasing cortical concentration of DA and 5-HT.	
	Olanzapine	CYP1A2 (CYP2D6)	Secondary actions with 5-HT1A, histamine, and adrenergic receptors	
	Risperidone	CYP2D6 (CYP3A4)	Olanzapine also antagonises muscarinic receptors	

NB: serotonin (5-HT), noradrenaline (NA), dopamine (DA).

^a^ Reversibile.

^b^ Bupropion is unicyclic; mirtazapine, mianserin, maprotiline, and amoxapine are tetracyclic.

^c^ https://pubchem.ncbi.nlm.nih.gov/pathway/PathBank:SMP0000641

^d^ https://s3-us-west-2.amazonaws.com/drugbank/fda_labels/DB11859.pdf?1553196718

^e^ https://www.fda.gov/drugs/science-and-research-drugs/table-pharmacogenomic-biomarkers-drug-labeling

?: Unknown metabolic pathway.

Though the *CYP3A* family is known to play a major role in the metabolism of ∼30% of all pharmacological drugs (Zemanova et al., 2022), the most well studied and important CYP genes in psychiatry are *CYP2D6* and *CYP2C19*. Both *CYP2D6* and *CYP2C19* are highly polymorphic genes with extensive interactions with the metabolic processes of tricyclic antidepressants (TCAs), selective serotonin reuptake inhibitors (SSRIs), and serotonin-noradrenaline reuptake inhibitors (SNRIs) (Müller et al., 2013; Shalimova et al., 2021; Zemanova et al., 2022). The interindividual genetic variations within these genes and the subsequently altered enzymatic and metabolic activity may explain some of the variation in drug response observed in people prescribed antidepressants. For example, of the more than 150 *CYP2D6* allelic variations designated to date by the Pharmacogene Variation Consortium (PharmVar)^4^ (Gaedigk et al., 2018)*,* over 40 variations are known to encode inactive or non-functional enzymes, with other variations encoding ‘normal’ or increased enzymatic activity (Bertilsson et al., 2002). Similarly, of the more than 30 *CYP2C19* variations have been designated by the PharmVar^4^, only seven have been reported to maintain normal enzymatic function (Gaedigk et al., 2018). Furthermore, *CYP2D6* and *CYP2C19* phenotypes vary greatly across the global population with studies suggesting that only 10% of people are poor metabolisers and even fewer (3%) are ultrarapid metabolisers (Martis et al., 2013; Gaedigk et al., 2017). With the implementation of PGx into clinical practice, it is possible to obtain *CYP2D6* and *CYP2C19* metaboliser status prior to prescribing antidepressants, accurately predicting dose and improving treatment outcomes (Altar et al., 2015; Arranz et al., 2019; Greden et al., 2019). Demonstrated in a recent study by Jukić et al. (2018) investigation into *CYP2C19-*encoded enzymes and their metabolism of escitalopram in 2,066 participants observed a noticeable difference in drug tolerability. Ultrarapid and poor metabolisers of *CYP2C19* were more likely to cease taking escitalopram due to both therapeutic failures in the case of ultrarapid metabolisers, as well as poor metabolisers presenting with ADRs. Jukić et al. (2018) conclude that by individualising escitalopram therapy and tailoring dosage dependent on *CYP2C19* status, PGx-guided treatment may limit the presentation of ADRs and improve therapeutic outcomes of escitalopram in clinical practice.

When investigating the interaction of *CYP2D6* and *CYP2C19* alleles with antidepressant metabolism in patients with depression, intermediate metabolisers have shown better responses to antidepressant medication with ultrarapid metabolisers presenting a higher risk of suicide than other metabolic phenotypes (Zackrisson et al., 2010). Additionally, poor metabolisers with reduced enzymatic activity, have shown higher residual concentrations of antidepressant levels in serum compared to extensive metabolisers, leading to adverse side-effects (Schenk et al., 2010; Huezo-Diaz et al., 2012; Chang et al., 2014; Milosavljevic et al., 2021). Finally, when prescribing antidepressants, it is pertinent to understand and recognise interactions with other drugs that are also metabolised on the *CYP2D6* and *CYP2C19* pathways to maximise therapeutic potential from all prescribed medications. For example, sertraline, a first-line SSRI, is metabolised by CYP enzyme CYP2B6 and at a dose of 50 mg it also acts as a mild inhibitor of CYP2D6 (Lynch et al., 2007). When dosage is increased from 50 to 200 mg, sertraline becomes a potent inhibitor, antagonising CYP2D6 and inhibiting the metabolism of other medications via this drug pathway, such as other common SSRIs (Sproule et al., 1997).

Research exploring drug-drug interactions in psychiatry and the involvement of CYP genetic variation has shown that polypsychopharmacotherapy increases a patient’s risk of potential adverse outcomes and hospital admissions, as well as having been retrospectively associated with an increased risk of suicidal behaviours (Goffin et al., 2016; Kessler et al., 2016). These retrospective studies have shown that individuals who have died of suicide had a greater likelihood of carrying more than two active copies of CYP gene *CYP2D6* than those dying of natural causes, thus corresponding to an ultrarapid metaboliser phenotype and failing to reach drug therapeutic potential (Zackrisson et al., 2010; Peñas-Lledó et al., 2012). Further studies associate not only high suicide risk with ultrarapid *CYP2D6* metabolism, but also implicate the involvement of ultrarapid *CYP2C19* metabolisers, and suggest that these phenotypes and polypharmacy factors need to be taken in account to prevent suicide attempt in patients with mental illness (Peñas-Lledó et al., 2022).

These studies, amongst others in the literature, demonstrate the benefit of implementing PGx testing for *CYP2D6* and *CYP2C19* metaboliser status in clinical practice. However, as PGx testing can currently provide information only on the probability of potential vulnerabilities, it should always be used in conjunction with clinical assessment (Namerow et al., 2020). Further research is suggested by most of the studies described above to advance the understanding of CYP involvement in psychiatric medication metabolism.

### 3.2 Pharmacogenetic testing in clinical psychiatric practice

It is not uncommon for people suffering from psychiatric conditions such as depression to persist through a trial-and-error process of therapies lacking efficacy to find the appropriate psychotherapeutic agents and dosages (Slomp et al., 2022). Nearly 60% of individuals suffering from depression are estimated to not completely respond to first-line antidepressants and around one in three people are predicted to not respond at all (Crisafulli et al., 2011). Furthermore, this same third of people treated for depression who do not show full remission following two or more treatment trials of first-line antidepressants may develop a persistent depressive disorder (Kverno and Mangano, 2021). This may be, in part, due to unsuitable medication and ADRs and possibly due to non-adherence to treatment recommendations after recurring failures in the trial-and-error treatment process (Howes et al., 2022). Furthermore, for each subsequent antidepressant prescribed to a person with depression, the likelihood of remission decreases significantly, with remission rates for the first and second drug trials at 36.8% and 30.6% respectively, and the third and fourth at 13.7% and 13.0% (Rush et al., 2006). PGx testing and informed prescribing has been suggested to improve treatment response and outcomes in those patients resistant to numerous antidepressants, decreasing the risk of drug-related side effects and patient non-compliance (Bousman et al., 2019).

As described above for *CYP2D6* and *CYP2C19*, many studies have explored the role of PGx gene polymorphisms in modifying psychiatric medication outcomes. However, there are few randomised controlled trials (RCTs) assessing the clinical utilisation of PGx testing as a predictive tool to guide prescription. Two RCTs published by Hall-Flavin et al. (2012); Hall-Flavin et al. (2013) found that patients who received PGx-guided treatment and tailored prescription had a significantly greater reduction in depressive symptoms after 8 weeks of antidepressant treatment when compared to those who received standard treatment with clinical guidelines. These studies utilised information from PGx CYP genes *CYP2D6*, *CYP2C19*, *CYP1A2*, as well as two genes encoding serotonin transporters (*SLC6A4*) and receptors (*HTR2A*). Similarly, two recent meta-analyses examining 1,737 participants over five RCTs and 5,347 participants over 11 RCTs, showed that PGx-guided treatment and tailored prescription and dosing were associated with higher remission rates and faster response times when compared to standard treatment in difficult-to-treat-depressive patients (Bousman et al., 2019; Wang et al., 2023). Vos et al. (2023) recently conducted a RCT exploring whether PGx-guided treatment would result in faster attainment of therapeutic TCA serum levels than standard of care treatment in 111 participants. They reported that participants receiving PGx-guided treatment realised therapeutic TCA concentrations faster than controls presenting with fewer and less severe side effects. No change was noted in depressive symptoms, however, indicating that whilst PGx-guided treatment was effective, perhaps future studies should investigate the use of second-generation antidepressants and not just TCAs.

Though the studies above suggest that PGx-guided treatment may be a promising approach to tailor drug treatments for patients with mental health disorders, there remain some ambiguities in the literature. Solomon et al. (2019) systematically reviewed 16 studies between 2013 and 2018 to investigate whether PGx testing of *CYP2D6* and *CYP2C19* was able to predict antidepressant response or ADRs. This study reported mixed findings, suggesting that PGx testing may predict ADRs in certain individuals, however, it was unclear if these results would translate across a broader population. Solomon et al. (2019) go on to mention that the lack of positive associations between PGx-guided treatment and reducing ADRs could range from explanations such as underpowered studies and the lack of ethnic diversity, to uncontrolled concomitant use of herbal medicines. Further RCTs should be conducted with adequate sample sizes to clarify whether CYP PGx-guided treatment can yield positive outcomes for mental health (Solomon et al., 2019).

In conclusion, current evidence from RCTs suggests that whilst PGx-guided treatment may aid in tailoring medication choice and dosing for patients with mental health disorders, further research is needed to fully understand the potential benefits of PGx-guided treatment and to address the challenges that currently limit its widespread use in clinical practice. It is also important to note that, although treatment with antidepressants has provided promising results for treating mental ill-health such as depression and anxiety, only 60%–70% of patients show an effective response to antidepressant therapy (Kennedy and Giacobbe, 2007; Al-Harbi, 2012). Further studies investigating the PGx profiles of patients who do not respond to medication may help guide selection of alternative treatments and identify the genetic profile of people with treatment-resistant depression (Fabbri et al., 2021; McCarthy et al., 2021).

## 4 Clinical application of pharmacogenetics in youth mental health

Mental ill-health in youth is a significant public health concern with the most recent Young Minds Matter survey estimating ∼600,000 Australian children and adolescents are currently living with some form of mental health disorder such as depression^5^ (Goodsell et al., 2017). Since the beginning of the COVID-19 pandemic, the prevalence of depression in youth significantly increased (Racine et al., 2021), with nearly two in five Australian people (39.6%) aged between 16- and 24-year reporting having mental ill-health in2020^6^. Additionally, a greater incidence of worry, loneliness, and depressive symptoms has been reported amongst Australian high school students since the pandemic outbreak in 2019 (Houghton et al., 2022). Although the situation has slightly improved since the end of 2021, the prevalence of mental ill-health in youth remains high, with many young people unable to access sufficient care and support for their mental health condition^7^. Depression in youth is associated with reduced quality-of-life outcomes such as school performance and social growth, as well as increases in the likelihood of risk-taking and self-harming behaviours (Goodsell et al., 2017). Adolescents suffering from untreated depression may continue to experience chronic depressive symptoms and impairment into their adult years, with a greater risk of developing a depression-related disability (Ghio et al., 2015). Furthermore, research findings indicate a correlation between chronic depression experienced during the ages of 12–17-year and subsequent psychosocial outcomes may affect individuals into their adulthood (Clayborne et al., 2019). These consequences include a higher likelihood of failing to complete secondary school, unemployment, and an increased in early/unwanted pregnancies.

With an increasing incidence of youth depression in recent years, lifetime healthcare costs covering mental health services such as psychiatric and other allied health professional services, hospitalisations and disability support services, and mental health-related subsidised prescriptions are also rising (Cook, 2019). Current lifetime costs associated with depression are AUD $43–70 billion/year to Australia’s economy^8^, encompassing not only healthcare costs, but also the loss of opportunity to the workforce through unemployment and/or loss of employment. Given the rising prevalence and socioeconomic burden attributed to mental health disorders in youth, there is a considerable need to reanalyse preliminary treatment options and the current care pathways to limit the mental health burden and to ensure that this population is receiving effective care (Le et al., 2021).

### 4.1 Guided antidepressant treatment in youth mental health

Although PGx-guided prescription is common in paediatric oncology and gastroenterology (Ramos et al., 2021; Sandritter et al., 2023), the majority of PGx studies to guide treatment for mental health have been conducted in adults with results extrapolated to youth populations (Wehry et al., 2018). Whilst there are limitations to PGx-guided treatment in youth, PGx testing has the potential to decrease morbidity, side effects and adverse event presentations, increase treatment response, and decrease hospital admissions, readmissions, and overall cost of care.

Studies observing metaboliser status and its association with escitalopram in children and adolescents echo those mentioned earlier by Jukić et al. (2018). Specifically, poor metabolisers for *CYP2C19* experienced more severe ADRs than other metaboliser statuses and have a high likelihood of discontinuing the use of escitalopram, citalopram, and sertraline (Aldrich et al., 2019; Poweleit et al., 2019). Furthermore, ultrarapid metabolisers tended to respond faster to both escitalopram and citalopram and subsequently spent less time in hospital following drug administration. These results reflect the observations of Strawn et al. (2019) who showed that differing *CYP2C19* metabolisers required differing doses of escitalopram and sertraline in order to maintain similar therapeutic benefits. *CYP2D6* polymorphisms have also been investigated in antidepressant prescription in youth. For example, fluvoxamine concentrations have been shown to remain higher for longer in *CYP2D6* poor metabolisers, with these patients therefore requiring dose adjustments (Chermá et al., 2011). Similarly, *CYP2D6* poor metabolisers are slower to metabolise fluoxetine into its active metabolite, norfluoxetine, than other metabolisers. As a result, at similar time points, fluoxetine concentrations are higher in poor metabolisers compared to other metabolisers (Gassó et al., 2014). A recent review conducted by Strawn et al. (2023) explored the acute and chronic ADRs often associated with SSRIs and SNRIs in children and adolescent patients. Strawn et al. discuss the presentation of acute gastrointestinal symptoms, long-term weight gain, and sexual dysfunction, suggesting that PGx-guided treatment may address these ADRs and inform discontinuation strategies from second-generation antidepressants in those youth patients presenting with symptoms.

Though the research above suggests PGx-guided treatment may be beneficial in guiding the treatment of mental ill-health in children and adolescents with antidepressant medication, not all studies have been as promising. Namerow et al. (2022), reviews a prospective trial investigating the use of PGx-guided treatment in adolescents with depression (Vande Voort et al., 2022). This trial randomised 176 adolescents with moderate to severe major depressive disorder to either receive PGx-guided treatment or treatment as usual, aiming to evaluate the clinical impact and potential for PGx testing panels to improve mental health outcomes in clinical practice in child and adolescent psychiatry. Results showed that there was no significant difference in symptom improvement, side effect burden, or satisfaction between the two groups. However, the study found that the use of PGx testing did influence clinical providers to more frequently prescribe medications that are not considered first-line antidepressants due to failed demonstrations in efficacy for the treatment of depression in youth. Namerow et al. (2022) go on to describe that whilst PGx testing may not improve treatment efficacy, there is growing evidence that PGx testing may be efficacious in specific medication prescription.

With mixed information surrounding the efficacy of PGx testing in youth for mental health disorders, it is vital to consider other factors of antidepressant therapy where PGx guided treatment may improve the current treatment model. When finding the right medication, one qualitative research study exploring young people’s views on medication reported that changing medication was often fraught with anxiety and that the switching of antidepressants was considered a frustrating and challenging process of trial-and-error, likely to increase medication non-compliance (McMillan et al., 2020). Recent research has shown that youth and the elderly, particularly those patients with depressive disorders, are at an extreme risk of lower medication adherence (Gast and Mathes, 2019). PGx provides an interventional tool to address these adherence issues. Though the evidence is limited, and further research is needed to critically analyse the effects of PGx-guided treatment on patient medication adherence, one study observed PGx testing was associated with a higher rate of adherence with 39 different medications (Christian et al., 2020). This study suggests that by prescribing more personalised medications using PGx testing and avoiding unsuitable and potentially high-risk drugs, genetically informed care may have a positive influence on medication adherence. Though this study assessed PGx-guided treatment in an adult population, this holds promise that personalised treatments in youth will help to increase adherence rates for mental health conditions and the use of psychiatric medication.

### 4.2 Challenges for pharmacogenetic implementation in youth mental health treatment

Whilst the research on PGx-guided treatment is promising, implementing patient-specific genetic testing to guide antidepressant prescription into clinical practice does not come without its challenges (Gurwitz and Motulsky, 2007; Pinzón-Espinosa et al., 2022). Precision medicine, or the individualised and tailored approach to medical treatment, has been made a government funding priority across the world, with countries such as the United States, Canada, the United Kingdom, and China making significant headway in their commitments to implementation over the last 5 years^9^ ^,^ ^10^ ^,^ ^11^. In 2018, Innovation and Science Australia, an independent board driving sustainable economic growth and societal benefits, identified an ideal National Mission to catalyse action around developing genetic-based precision medicine tools to customise care for each individual by 2030 and provide the right treatment first^12^. However, this policy, as well as other scientific research (Mutsatsa and Currid, 2013; Amato et al., 2018; Corponi et al., 2018; Herbert et al., 2018; Corponi et al., 2019; Goodspeed et al., 2019; Vest et al., 2020; Jameson et al., 2021; Ramsey et al., 2021), note significant barriers that need addressing to facilitate the adoption of precision medicine practices into routine clinical practice. In the context of PGx-guided treatment, even if the translation of PGx testing utilisation to personalise medicine in psychiatry has accelerated, until these barriers are addressed, PGx testing will likely remain research-use only (Bousman et al., 2017; Blasco-Fontecilla, 2019).

#### 4.2.1 Lack of clinical guidelines for youth

Currently, there are limited evidence-based guidelines for the use of PGx in youth mental health (Abdullah-Koolmees et al., 2020), causing a lack of consensus amongst primary care clinicians on the best way to apply genetic testing to medication selection and dosing (Gizer et al., 2009). Though the Clinical Pharmacogenetics Implementation Consortium (CPIC) recently provided guidelines on the interpretation of PGx testing for psychiatric medication (Hicks et al., 2017; Bousman et al., 2023), it is noted that children and adolescents were underrepresented in many of the studies of the drugs listed in these guidelines. The CPIC therefore recommends that the generalisability of recommendations to paedeatric patients needs to be established. Furthermore, many of the PGx trials in youth psychiatry have been small and not yet replicated, limiting the applicability of their findings to govern these CPIC guidelines. However, recent research may suggest that adult data can be used to govern the guidelines for child and adolescent PGx-guided treatment. Paediatric pharmacology has traditionally held the idea that children are not small adults (Stephenson, 2005). Children undergo phases of rapid growth and development, and their bodies and organ systems function differently than those of their adult counterparts. Considering this, it has always been thought that pharmaceuticals may exhibit different effects and safety profiles in paediatric patients, resulting in the requirement for different prescriptions and dosages. However, studies in pharmacokinetics suggest that in this domain, children may indeed be treated as small adults (Stephenson, 2005; Anderson and Holford, 2013). Though controversial, Anderson and Holford explain how adjusting adult pharmacological doses using a demographic covariate of size, maturation, and organ function can scale doses from adults to children (O'Hara, 2016). If possible, this then begs the question: do we require separate evidence of efficacious PGx-guided treatment in youth if evidence exists in adults (Barker et al., 2022)? Stephenson reports that the perception of variable responses in children compared to adults arises because drugs are not adequately studied in youth populations of different ages (Stephenson, 2005). Stephenson continues to discuss how the response to drugs in youth have much in common with the responses observed in adults. With the majority of PGx research conducted in adults and extrapolated to youth, the extent to which we can rely on adult PGx to safely dose medication in youth needs to be established. Therefore, more age-appropriate RCTs with a youth focus using PGx-guided treatment in clinical practice are required to compare to their adult counterparts to build a robust evidence-based guideline for mental health treatment (Brothers, 2013; Van Driest and McGregor, 2013).

#### 4.2.2 Experience and attitudes of general practitioners

General practitioners (GPs) are often the first level of contact in the Australian healthcare system, with mental health related issues representing one of the most common health issues managed by GPs (Carey et al., 2020). However, implementing PGx-guided mental health treatment poses many challenges to primary healthcare providers–these include the limited knowledge and experience of PGx testing, the evidence basis for its use, and the concerns about how it can be incorporated into current workflow (Vest et al., 2020; Jameson et al., 2021). As a relatively new field in standard medical practice, many healthcare professionals lack the training and expertise to interpret PGx testing results and use them to inform treatment decisions (Prows and Saldaña, 2009). Many psychiatric medications are prodrugs, meaning they are prescribed as inactive derivatives that when metabolised by CYP enzymes, convert into active compounds (Lynch et al., 2007). Conversely, some drugs, including numerous SSRIs, are administered as active compounds that require deactivation by CYP enzymes during the drug metabolising process. Having a comprehensive knowledge of common psychiatric drugs is paramount when properly interpreting PGx testing information to ensure patient safety and avoid ADRs from drug-drug interactions. A recent review by Ong et al. (2022) investigated the experience, attitudes, and knowledge of GPs towards the application of PGx in primary practice and found that whilst most GPs understood the basic principles and recognised the potential benefits of using PGx testing, many were uncertain about how to use genetic testing to inform treatment decisions. This was further supported in recent findings, where the implementation of PGx education and training efforts were shown to improve the comfort felt by primary care providers, such as GPs, when ordering a PGx test for their patients (Preys et al., 2023). These encouraging results demonstrate an enthusiasm for PGx-guided treatment in GP practices and welcome the idea that with proper support, guidance, and education, primary care providers may implement PGx testing into their clinical practice.

Although the studies above illustrate a keen understanding on the usefulness for PGx testing in the primary care setting, with no endorsed guidelines on the use of PGx testing in clinical practice, many GPs have expressed the need for further research that explores the translation of new genetic technologies into clinical practice to help inform the development of such guidelines. The review by Ong et al. (2022) emphasised the importance of gathering a complete understanding of the experiences, knowledge, and attitudes held by GPs towards genetic testing. Gathering this information and tailoring the integration of PGx testing to the requirements of primary care providers is integral for successful adoption into clinical practice (Aboelbaha et al., 2023).

#### 4.2.3 Attitudes and expectations of young people with mental ill-health

As well as those of GPs, the knowledge and attitudes of the patient towards PGx testing are reported in the literature. Stancil et al. (2021) explored adolescent perceptions of PGx testing and noted the lack of research on this topic. This study emphasised the importance of further research exploring youths’ perceived values and understandings of PGx testing, as well as strategies on disseminating PGx results to youth. All participating youth in this study were optimistic for the implementation of PGx-guided treatment in clinical care, expressing an understanding for its use in primary care, the low risk associated with testing, and the benefit for them and their peers in their treatment (Stancil et al., 2021). Stancil et al. conclude by noting the importance for including youth in the decision-making process and engaging them in the discussion of PGx testing results and the relevance of these results to the medication they may be prescribed.

#### 4.2.4 Societal expectations

Lastly, societal expectations around data privacy, equity, and economic values may provide a hurdle in normalising PGx-guided treatment^12^. Public awareness around data privacy has increased in recent years and has become a sensitive issue in the healthcare sector (Vimalachandran et al., 2020). Though there are many benefits to PGx testing, the broader community will only have confidence in the use of genetic data if privacy can be guaranteed. Precision medicine tools and applications need also to be available to all peoples, including people of different ethnicities and those living in remote communities^12^. Finally, studies have found that a large concern for both patients and healthcare professionals is the cost of PGx testing (Jameson et al., 2021). Multiple studies have found that costing was the largest factor influencing patients’ decision making (Chan et al., 2017; Liko et al., 2020), highlighting that patients would be more likely to seek PGx testing if the cost was lower or subsidised by Medicare.

To navigate the barriers and educate the public on the potential benefits of PGx-guided treatment for improved mental health outcomes in youth, it is important that researchers engage with the communities that will benefit from the adoption of this technology.

## 5 Conclusion

Mental health conditions are becoming increasingly prevalent in Australian society and throughout the Western world, particularly in our youth. The management of mental health conditions in youth is complex and, if not handled correctly, can exacerbate mental ill-health and risk the persistence of these conditions into adulthood. Although there have been improvements in symptom recognition, diagnostic guidelines, and the availability of pharmacotherapies, there remain many challenges in the management of mental health in young people. PGx testing has been shown to provide an increased chance of achieving a therapeutic outcome, minimising harmful ADR and their flow on effects. Current research suggests that an informed knowledge of youth’s specific PGx characteristics could be expected to enhance the treatment and recovery from mental illness. Although there are barriers to the use of genomic testing in youth, the field of PGx continues to develop and advances in this area have led to an increased accessibility of DNA testing and PGx-guided treatment. Whilst emerging literature has shown PGx testing may be utilised in the care of young people with mental illness, there is a need for more large-scale age-appropriate studies to resolve conflicting research. In addition, further studies are needed to explore how this testing could be incorporated into standard clinical practice, navigating social, behavioural, and economic barriers. With most mental illness in Australia managed in primary care, it is important that the application of PGx testing in youth is extensively studied in the context of primary healthcare settings. Therefore, future research in PGx-guided treatment needs to acknowledge the experiences of youths themselves and the concerns of their primary care providers, involving key stakeholders in the research process to ensure personalised and effective care.

## Footnotes

1
https://news.vumc.org/2020/04/30/predict%E2%80%88program-expands-opens-new-genomics-clinic/

2
https://www.psa.org.au/wp-content/uploads/2019/01/PSA-Medicine-Safety-Report.pdf

3
https://vizhub.healthdata.org/gbd-results/

4
https://www.pharmvar.org/

5
https://www1.health.gov.au/internet/main/publishing.nsf/Content/mental-pubsm-child2

6
https://www.abs.gov.au/statistics/health/mental-health/national-study-mental-health-and-wellbeing/latest-release

7
https://psychology.org.au/for-members/news-and-updates/news/2022/australians-need-psychological-help-more-than-ever

8
https://apo.org.au/node/309475

9
https://www.genomicsengland.co.uk/initiatives/100000-genomes-project

10
https://allofus.nih.gov/

11
https://www.weforum.org/agenda/2017/11/3-ways-china-is-leading-the-way-in-precision-medicine/

12
https://www.chiefscientist.gov.au/sites/default/files/Precision-medicine-final.pdf
